# Authors’ Response to Peer Reviews of “Continuous User Experience Monitoring of a Patient-Completed Preoperative Assessment System in the United Kingdom: Cross-sectional Study”

**DOI:** 10.2196/35504

**Published:** 2022-01-06

**Authors:** Inocencio Daniel Maramba, Arunangsu Chatterjee

**Affiliations:** 1 Centre for Health Technology University of Plymouth Plymouth United Kingdom

*This is the authors’ response to peer-review reports for the paper “Continuous User Experience Monitoring of a Patient-Completed Preoperative Assessment System in the United Kingdom: Cross-sectional Study.”*

## Round 1 Review

### Reviewer BG

#### General Comments

Thank you for your comment [1]. We also feel that user experience evaluation and feedback is important in the development of eHealth applications.

#### Specific Comments

##### Minor Comments

1. Thank you for your comment. We sought to introduce this innovation [2] to prevent “questionnaire fatigue.”
2. Thank you for your comment. We have included that in the Conclusions and Recommendations section.

### Reviewer CZ

#### General Comments

Thank you for your very insightful and helpful comments. Our main points were summarized properly. We have followed your recommendations regarding the title, the Abstract, and the Methods and Discussion sections. The specific changes we have made are outlined below.

#### Specific Comments

Thank you for your comments [3]. We have implemented the recommendations as specified in the responses to the comments below.

##### Major Comments

1. Thank you for your comment. We have changed the title to the one suggested.
2. Thank you for your comment. We have followed your suggestions and revised the abstract accordingly.
3. Thank you for your comment. We have followed your suggestions and revised the abstract accordingly.
4. Thank you for your comment. We have structured the introduction as recommended.
5. Thank you for your comment. We have moved the last paragraph to the *Rationale* section.
6. Thank you for your comment. We have restructured the *Methods* section as outlined.
7. Thank you for your comment. We have added text justifying the use of the statistical tests.
8. Thank you for your comment. We have organized the data analysis subsection as suggested.
9. Thank you for your comment. We have included the suggested guideline and included the checklist as a Multimedia Appendix.
10. Thank you for your comments. We have organized the *Results* section as suggested.
11. Thank you for your comments. We have organized the *Results* section as suggested.
12. Thank you for your comment. For the continuous variables, we tested the differences between groups using both parametric (*t* test, analysis of variance) and nonparametric tests (Wilcoxon rank sum, Kruskal-Wallis). The Shapiro-Wilk test for both age and completion times showed that the data were not normally distributed. We have revised the table to report the median ages and completion times.
13. Thank you for your comment. We have revised the tables to report the medians.
14. Thank you for your comment. We have corrected the terms as suggested.
15. Thank you for your comment. We will report the median completion times, as the data were not normally distributed according to the Shapiro-Wilk test.
16. Thank you for your comment. We have merged the tables as suggested.
17. Thank you for your comment. We have added that paragraph to the *Results* section.
18. Thank you for your comment. We have organized the Discussion as suggested above.
19. Thank you for your comment. We have moved the ethical considerations to the *Methods* section.
20. Thank you for your comment. We listed the appendices before the references and moved the abbreviation list to the end of the paper.

##### Minor Comments

1. Thank you for your comment. We have done so.
2. Thank you for your comment. We have amended the Abstract accordingly.
3. Thank you for your comment. We have modified the statement as suggested.
4. Thank you for your comment. We have formatted the tables according to the guidelines.
5. Thank you for your comment. We have merged some of the tables and so decreased the number of tables to 5.
6. Thank you for your comment. We have reformatted the *P* values to follow the guidelines.
7. Thank you for your comment. We have restructured the *Conclusion* as suggested.
8. Thank you for your comment. We have followed the suggestion given.
9. Thank you for your comment. We have reformatted the references in AMA citation style and included PMIDs and DOIs where available.
10. Thank you for your comment. We have traced the pdfs of all the articles.

## Round 2 Review

### Reviewer CZ

RESPONSE: Thank you for your comment. We have followed your suggestion and used the text in the Results section of the Abstract as the “Principal findings” and moved the rest of the text in the discussion to under “Comparisons with prior studies.” We have also added some more information about how our results compare favorably with prior studies.

We would like to thank the reviewers for their very insightful and helpful comments that greatly aided in improving this paper.
